# Physical activity level, fear of falling and quality of life: a comparison between community-dwelling and assisted-living older adults

**DOI:** 10.1186/s12877-020-01982-1

**Published:** 2021-01-06

**Authors:** Christopher Olusanjo Akosile, Charles Kenechukwu Igwemmadu, Emmanuel Chiebuka Okoye, Adesola Christiana Odole, Ukamaka Gloria Mgbeojedo, Ayodeji Ayodele Fabunmi, Ifeoma Uchenna Onwuakagba

**Affiliations:** 1 Department of Medical Rehabilitation, College of Health Sciences, Nnamdi Azikiwe University, Nnewi Campus, Nnewi, Anambra State Nigeria; 2 Department of Physiotherapy, College of Medicine, University of Ibadan, Ibadan, Oyo State Nigeria; 3 Department of Medical Rehabilitation, Faculty of Health Sciences and Technology, University of Nigeria, Enugu Campus, Enugu, Nigeria

**Keywords:** Physical activity, Fear of falling and quality of life, Comparison, Older adults, Community-dwelling, Assisted-living facilities

## Abstract

**Background:**

Physical activity (PA), fear of falling (FOF) and quality of life (QOL) are very important constructs in geriatrics. The interplay among these constructs may vary between community-dwelling and assisted-living older adults. However, studies comparing the wellbeing of community-dwelling older adults with those residing in the assisted-living facilities (ALFs) are rather rare especially from developing countries. This study was aimed at comparing PA, FOF and QOL between assisted-living and community-dwelling older adults and also determining the correlations amongst the constructs for each group.

**Methods:**

This cross-sectional survey involved consecutively sampled 114 older adults (≥65 years, ambulant and well-oriented in time, place and person) residing in conveniently selected ALFs (11.3% males) and adjoining communities (54.1% males). PA, FOF and QOL were evaluated using the Physical Activity Scale for the Elderly, the Modified Fall Efficacy Scale and the Short-form Health Survey (SF-36) questionnaire respectively. Data was analysed using descriptive statistics, analysis of covariance and Spearman rank-order correlation test at 0.05 level of significance.

**Results:**

Participants from the ALFs had significantly lower domain and overall PA (*F*=5.6–103.34; *p*< 0.05) and QOL (*F*=11.12–118.05; *p*< 0,05) scores than community-dwelling groups. FOF was significantly more prevalent in assisted-living group (*p*< 0.05). There were significant positive correlations (*p*< 0.05) between each pair of PA, FOF and QOL for both assisted-living and community-dwelling groups.

**Conclusions:**

Older adults in the ALFs had lower PA and QOL scores with higher prevalence of FOF than their community-dwelling counterparts. Significant relationships existed between PA, FOF and QOL for participants in either group. Present results may be suggesting that ageing in place ensures better health outcomes than institutionalised ageing. Whenever possible, older adults should therefore be encouraged to age in place rather than moving into ALFs.

## Background

Regular physical activity (PA) is very essential for healthy living and ageing [1]. Among older adults, active lifestyles aid in the maintenance and promotion of health and well-being [2–4], and in reducing the need for care and hospitalisation and the risk of mortality and premature death [3]. It also contributes to the primary and secondary prevention of several chronic diseases (including cardiovascular diseases, cancer, hypertension and obesity) [2, 3], and the enhancement of social interactions, mobility and cognitive performance [3]. On the other hand, physical inactivity can lead to: high morbidity, substantial economic burden and early death [5, 6]. Despite the beneficial effects of PA and the adverse effects of physical inactivity, majority of older adults do not meet the recommended levels of PA (engaging in 150 min a week of moderate-intensity, or 75 min a week of vigorous-intensity PA, or an equivalent combination of moderate-and vigorous-intensity PA) [7–9]. This could be attributed to several factors including demographic factors, available social support and facilities for PA, motivation, self-efficacy, physical limitation, falls, and so on [10, 11].

Some older adults disengage from physical, social or leisure activity after having experienced a fall [12, 13]. This disengagement may be due to fear of falling (FOF) [12] which may be influenced by physical, psychological or functional factors [14–16]. A previous study had shown older individuals with FOF to have poorer PA level than those without FOF [12]. Individuals who exhibited FOF also had lower scores in all the quality of life (QOL) domains (comprising physical functioning, role imitation due to physical health, bodily pain, general health perception, vitality, social functioning, role limitation due to emotional problem and general mental health) compared to those without FOF in the same study [17].

Quality of life (QOL), defined as the degree of enjoyment and individual’s ability to function in the community and interact with the society [18], is determined by several factors [19]. Other than socio-demographic features such as age and financial status, health (including functional disability), and social support and networks are often found to be important in older adults’ ratings of their QOL [19, 20]. In general, the common observation is that older adults value social integration, independence, emotional support and financial security [19, 21–23].

In most developed countries, the steady rise in the numbers and proportion of older adults had led to instituting other housing options like assisted-living facilities (ALFs) and nursing homes for the older adults. These facilities cater for older adults whose chronic physical and cognitive morbidities necessitate social and health services that are neither available nor can be feasibly provided at the community setting [24]. However, some circumstances (like difficulty adjusting to new living environment and the feeling of loss of control over personal decision making) can truncate the ultimate goals of these facilities, thereby making older adult residents’ lives more difficult [25–27]. Consequently, there seems to be a form of disagreement on which one is better between keeping older adults in the community and allowing them to age in place rather than sending them to specialised institutions [28]. In Nigeria and other developing nations within and outside Africa, ALFs are not socially and culturally acceptable [29, 30]. The available ones are run by missionary non-governmental organisations that are often not well-funded to be able to provide the facilities and level of care needed as obtained in the developed world. However, with the continuous explosive increase in the population of older adults especially in developing countries, there is every likelihood of increased patronage of ALFs in these countries. There may therefore be need to compare health outcomes (like QOL, FOF, and PA) between assisted-living and community-dwelling older adults. This may help to identify some of the areas of needs of these ALFs so as to be able to proffer appropriate solutions.

Studies comparing the wellbeing of community-dwelling older adults with those residing in the ALFs are rather rare especially from developing countries. This also seems to be the case in developed countries where previous studies had only compared the health status of older adults before and during their living in ALFs [27]. While ageing in place is desirable, it may not always be practicable for some groups of older adults. For such, it will be important to make the ALFs where they reside as homely as possible. Evidence from literature suggests that some exercise interventions against FOF found to be effective in the community setting were not effective in the assisted-living setting and vice-versa. This then suggests that differences in the interplay between FOF and PA exist across settings. Considering the fact that typical African society is still largely averse to the idea of living in ALFs, the psychological burden for residents in such facilities could only be imagined. We therefore hypothesized that residents of ALFs would report poorer health outcomes than their community-dwelling counterparts. This study was therefore designed to find out the levels, relationships and comparisons of FOF, QOL and PA between older adults residing in ALFs and those residing in the surrounding communities.

## Methods

### Design

This was a cross-sectional survey involving consecutively recruited volunteering older adults aged 65 years and above residing in three conveniently selected ALFs and their surrounding communities in selected cities in Anambra and Enugu States of Nigeria. The ALFs were selected because of their proximity to the researchers. The participants were ambulant fallers or non-fallers who were well-oriented in time, place and person. One ALF each in cities of Nnewi and Onitsha (Anambra State) and Enugu (Enugu State) were used for this study. Before the commencement of this study, ethical approval was sought and obtained from the Ethical Review Committee of Nnamdi Azikiwe University Teaching Hospital (NAUTH/CS/66/VOL.8/099). Permission was sought and obtained from the community heads and directors of the ALFs in order for the study to be carried out in their domains and also for them to help mobilise the older adults that were resident in their respective communities and institutions. Informed consent was sought and obtained from the participants after the aims and procedures of the study had been explained to them. The volunteers were then consecutively recruited for the study in the ALF, individual homes or community centres. Information on participants’ socio-demographic characteristics was sought and obtained through oral interview. The Physical Activity Scale for the Elderly (PASE), the Modified Fall Efficacy Scale (MFES) and the Health Survey 36-Item Questionnaire (SF-36) were used to measure the participants’ levels of PA, FOF and health-related QOL respectively. The instruments were self-administered to participants who could read and understand English Language. The cross-culturally adapted and validated Igbo versions of the instruments [[31, 32], unpublished data] were interviewer-administered to those participants who could not understand English Language. It took 35–45 min to administer the three questionnaires to each older adult.

### Instruments

#### Physical activity scale for the elderly (PASE)

The PASE is a 12-item reliable and valid instrument designed specifically to assess PA in older persons over a one-week time frame. It comprises of items regarding the frequency and duration of leisure activity (e.g., sports, jogging, swimming, strengthening and endurance exercise), household activity, and work-related activity during the previous 7-day period. Participation in leisure-time and strengthening activities are scored as never, seldom (1–2 days per week), sometimes (3–4 days per week), and often (5–7 days per week). Duration of these activities is scored as less than 1 h, 1–2 h, 2–4 h and more than 4 h. Household and work-related activities are scored as yes or no. In work-related activities, paid or unpaid work is scored in hours/week. The total PASE score is computed by multiplying either the time spent in each activity (hours per week) or participation (i.e., yes/no) in an activity, by empirically derived item weights and then summing overall activities. The overall PASE score ranges from 0 to 400 or more and higher scores reflect higher PA level [33–35].

#### Modified fall efficacy scale

The MFES is a reliable and valid 14-activity questionnaire that is an expanded version of the original 10-activity Fall Efficacy Scale (FES). The MFES included outdoor activities which the FES does not cover. Each item on MFES was scored on the 10-point visual analogue scale with “0” denoting “not confident or not completely sure”; “5” signifying “fairly confident or fairly sure”; and “10” meaning “completely confident or completely sure.” Scores can fall in between 0, 5 and 10. Higher scores reflect more confidence and less FOF while lower scores reflect less confidence and more FOF [36]. The MFES is the only instrument for assessing FOF that has been cross-culturally adapted and validated for use in Igbo (Nigerian) environment [32].

#### Health survey 36-item questionnaire (SF-36)

The SF-36 is a 36-item, self-administered, valid, reliable and widely used questionnaire for measuring health-related QOL. It has eight domains, two composite dimensions and a total score. The domains are physical functioning, role limitation due to physical health, bodily pain, general health perception, vitality, social functioning, role limitation due to emotional problem and mental health. The first five domains make up the “physical health” dimension while the last five form the “mental health” dimension. The vitality and general health domains are parts of both dimensions. Hence, each dimension includes three specific and two overlapping scales [37]. Domain, dimension and total scores (which are got by finding the average scores of the responded constituting items) range from 0 to 100, with a higher score indicating a better health related QOL.

### Data analysis

Data was analysed using the SPSS (version 21). The socio-demographic, PA, FOF and health-related QOL data were analysed using the descriptive statistics of frequency count, percentage, mean and standard deviation (SD). Data were tested for normality using the Kolmogorov-Smirnov’s test. T-test and Chi-square test were used to examine differences in sample characteristics between assisted-living and community-dwelling participants. Analysis of covariance was used to compare FOF, PAs (domain and total scores) and health-related QOL (domain and total scores) between older adults living in the community and those in ALFs while controlling for their age, gender, marital status and literacy level. Spearman rank-order correlation test was used to determine the relationships amongst PA, FOF and health-related QOL in each group. Alpha level was set at 0.05.

## Results

### Sample characteristics

One hundred and fourteen (114) older adults comprising 53 (46.49%) residents of ALFs (mean age=78.98±9.36 years) and 61 (53.51%) community-dwellers (mean age=71.28±7.85 years) participated in this study. The assisted-living older adults were significantly older (*t=4.28; p< 0.001*), and had significantly higher proportion of females (***χ***^2^=*23.06, p< 001*), less literate participants (***χ***^2^=*5.59, p=0.020*) and more widows and widowers (***χ***^2^=*33.50, p< 0.001*) than those in the communities (Table 1).

**Table 1:** Socio-demographics distributions of participants

Variable	Class	Assisted-living facility (***n***=53) f (%)	Community-dwelling (***n***=61) f (%)	***χ***^2^(***n***=114)	***p***
Gender	Male	6 (11.3)	33 (54.1)	23.06	< 0.001*
	Female	47 (88.7)	28 (45.9)		
Marital status	Single	0 (0)	2 (3.3)	33.50	< 0.001*
	Married	8 (15.1)	40 (65.6)		
	Widowed/ Divorced	45 (84.9)	19 (31.1)		
Occupation	Active	0 (0)	31 (50.8)	37.00	< 0.001*
	Inactive	53 (100)	30 (49.2)		
Literacy level	Literate	17 (32.1)	33 (54.1)	5.59	0.020*
	Non-literate	36 (67.9)	28 (45.9)		

KEY

*= significant at *p*< 0.05

Active = Having some form of occupational engagement predominantly trading/farming.

Literate = Could read and/or write either in English or Igbo Language.

### Levels of FOF, PA and QOL between community-dwelling and assisted-living older adults

Prevalence of FOF among assisted-living and community-dwelling participants were 100.0 and 1.6% respectively. The total PASE scores for the assisted-living and community-dwelling older adults were 11.84 and 152.58 respectively. The total QOL scores for the assisted-living and community-dwelling participants were 44.21±11.41 (poor) and 75.05±11.81 (good) respectively. The participants from the ALFs had their best score in the emotional function domain (61.58±9.96) and poorest scores in the physical function, the role limitation due to physical problems and the role limitation due to emotional problems domains (26.51±20.01). Conversely, community-dwelling participants had the best score in two of the three domains (physical function and role limitation due to emotional problems domains) that the assisted-living participants had their poorest score (Table 2). For participants in the ALFs, the major contributors to their PASE scores were walking, light household activity and caring for another person whereas the PASE score of the community-dwelling participants was mainly from light household activity, walking and paid/unpaid work. Contributions were generally low in sports/leisure time related domains for both groups. (Table 3).

**Table 2:** Analysis of covariance showing differences in quality of life scores between assisted-living and community-dwelling elderly

Variables	ALF (***n***=53) Mean±SD	CD (***n***=61) Mean±SD	***F*** (***n***=114, df=1)	***p***-value
Physical function	26.51±20.01	79.43±20.19	118.05	< 0.001*
RLPF	24.06±39.82	67.21±37.51	15.28	< 0.001*
RLEP	35.22±44.54	91.80±24.08	41.86	< 0.001*
Vitality	48.77±9.75	58.26± 14.59	11.12	0.001*
Emotion function	61.58±9.96	77.28±7.81	46.93	< 0.001*
Social function	54.76±20.62	79.10±15.43	22.08	< 0.001*
Pain	55.38±23.91	79.14± 18.62	14.64	< 0.001*
General health	53.58±12.41	68.36±11.86	2.07	< 0.001*
MCS	47.90±9.56	73.52±9.54	111.79	< 0.001*
PCS	40.52±14.58	76.59±14.81	96.19	< 0.001*
Total QOL	44.21±11.41	75.05±11.81	113.37	< 0.001*

KEY

Age, gender, marital status and literacy level were controlled for.

*ALF* Assisted-living facility.

*CD* Community-dwelling.

*: significant at p< 0.05

*RLPF* Role limitation due to physical problem domain score.

*RLEP* Role limitation due to emotional problem domain score.

*MCS* Mental component score.

*PCS* Physical component score.

*QOL* quality of life

**Table 3:** Analysis of covariance showing differences in PASE scores between assisted-living and community-dwelling older adults

Variables	ALF (***n***=53)	CD (***n***=61)	***F*** (***n***=114; df=1)	***p***-value
Walk	5.14±5.41	47.09±52.10	15.88	< 0.001*
Light sport (e.g. bowling)	0.48±1.92	3.90±5.16	9.35	< 0.010*
Moderate sport (e.g. double tennis, ballroom dancing)	0.09±0.68	1.44±2.82	2.23	0.140
Strenuous sports (e.g. jogging, swimming)	0.00±0.00	0.81±2.23	8.41	0.010*
Muscle strength and endurance	0.00±0.00	1.00±3.29	6.31	0.010*
Light house work (e.g dusting or washing dishes)	3.77±9.04	23.36±6.24	103.34	< 0.001*
Heavy house work (e.g. scrubbing floors, washing windows)	0.47±3.43	14.75±12.40	38.37	< 0.001*
Home repair	0.00±0.00	16.72±15.02	30.16	< 0.001*
Outdoor gardening	0.00±0.00	10.49±10.07	41.97	< 0.001*
Lawn work	0.00±0.00	18.89±18.13	31.38	< 0.001*
Care for others	1.32 ±6.73	12.62±16.94	19.50	< 0.001*
Volunteer work	0.40±2.88	2.75±7.14	5.06	0.030*
PASE total	27.15±28.53	190.82±110.58	60.59	< 0.001*

KEY

PASE=Physical Activity Scale for the Elderly.

*=significant at p< 0.05

*ALF* Assisted-living facility.

*CD* Community-dwelling.

Age, gender, marital status and literacy level were controlled for.

### Comparisons of FOF, PA and QOL between community-dwelling and assisted-living older adults

Residents of ALFs had significantly more FOF than their community-dwelling counterparts. Participants living in the ALFs had significantly lower total and domain QOL scores than the community-dwelling participants after controlling for participants’ age, gender, marital status and literacy level (*F*=*11.12–118.05; p< 0.050*) (Table 2). After age, gender, marital status and literacy level were statistically controlled for, participants in the ALFs had significantly lower fall efficacy score (*F*=*159.17; p< 0.001*) and total and individual domain PA scores than the community-dwelling participants (*F*=*5.06–103.34; p< 0.050*) except in the moderate sport domain which was still higher among participants residing in the communities (Table 3).

### Correlational analyses

Following the non-normality in the data distribution, Spearman rank test was employed in determining the correlations between FOF, PA and QOL. There was a significant and positive relationship between each pair of PA, FOF (falls self-efficacy) and QOL among both the assisted-living participants and those in the communities with each predicting between 7 to 31% variances in the order. The relationships between PA and FOF were higher in assisted-living than in community-dwelling participants, whereas the relationships between QOL and each of PA and FOF were higher for community-dwelling than for assisted-living participants (Table 4).

**Table 4:** Spearman rank-order correlations between physical activity, falls self-efficacy and quality of life among the participants

Variable	ALF (***n***=53) (***r***- value)	CD (***n***=61) (***r***- value)
Physical Activity and FOF	*r*= 0.518*	0.266*
	*p*=0.000	0.038
	*cd*=0.27	0.07
Physical activity and Quality of life	*r*= 0.316*	0.427*
	*p*=0.021	0.001
	*cd*=0.10	0.18
FOF and quality of life	*r*= 0.534*	0.556*
	*p*=0.000	0.000
	*cd*=0.29	0.31

KEY

*=significant at *p*< 0.01

*cd* Coefficient of determination.

*FOF* Fear of falling.

*ALF* Assisted-living facility.

*CD* Community-dwelling.

## Discussion

### Sample characteristics

This study was aimed at determining and comparing the levels of PA, FOF and QOL among community-dwelling older adults and those in the ALFs in selected cities in Anambra and Enugu States of Nigeria. In the present study, ALF older adults had significantly higher FOF and lower PA and QOL than the community-dwelling group. Significant relationships existed between variable pairs (among PA, FOF and QOL) for participants in either group with the relationships between PA and FOF being higher in ALF group, while the relationships between QOL and each of PA and FOF being higher in the community-dwelling group. Unlike in the community, more women (88.7%) than men were residing in the ALFs. Hsu & Jhan [38] reported that more male than female older adults usually have their spouses still alive, such that the women are more likely to require institutionalisation. The finding of more female widowed participants in the ALFs in the present study seems to support Hsu & Jhan [38] report. In the present study, 58 % (58%) were still occupationally active similar to findings in the population sampled by Horowitz & Vanner [27]. None of the older adults in the ALFs was occupationally active probably for lack of opportunity in the facilities.

### PA levels of the participants

The community-dwelling older adults in the present study were very physically active with a mean PASE score of 146.77. This score is higher than values obtained from previous local [12] and European [39] studies. The fact that the study participants in the Akosile et al. [12] study were slightly older and had fewer occupationally active participants compared to this study could have resulted to this difference. Community-dwelling older adults in the present study were significantly more physically active than their counterparts in ALFs. This may be due to higher proportion of relatively younger and more occupationally active older adults in the community compared to their counterparts in ALFs. Major contribution to the PASE score among community-dwelling older adults were light housework, walk activity, paid/unpaid work and lawn work or yard care which is in line with a previous report [12]. Good contributions from light housework, lawn work or yard care are to be expected as older adults in most communities tend to be more involved in household related activities. It seems however that walk activity was helpful in enhancing PA status among older adults in the community as shown by findings from the present study. This may invariably be helpful for achieving and maintaining good health. It is also worth noting that in this study, sport activities (light sport, moderate sport, strenuous sport and muscle strength/endurance) constituted a very low proportion of the total activity level. This is also in line with the findings of Akosile et al. [12]. Participants’ level of awareness on the relevance of sport activity to improving their QOL was not assessed in this study but may have contributed to low levels of sports activity participation in the group. Though major contribution to the PASE score among older adults in ALFs were walking, light housework and caring for another person, only a few residents actually engaged in these activities culminating in the very low total score for all domains. It is also worth noting that household-related activities and sport activities constituted a very low proportion of total activity level. The generally low PASE scores across domains may be because care providers in the ALFs were practically providing assistance in all activities of daily living, hardly allowing residents to do things by themselves.

### QOL levels of the participants

Older adults in the ALFs had lower QOL scores than those dwelling in the community consistent with a previous finding [27]. This may not be unconnected to the socio-cultural belief among Africans that children or extended family members are meant to take care of ageing or ailing individuals [29, 40] thereby making the dwellers of ALFs to feel less fulfilled with resultant adverse impediment of their QOL. Furthermore, most of the participants in the ALFs had several impairments that could limit their activity such that they would require assistance for their daily activities. However, since the present study did not set out to investigate this, it is unknown to what extent these observed conditions contributed to reduction in QOL among the group. Older adults in the ALFs had their best QOL score in the emotional function domain and poorest scores in the physical function, the role limitation due to physical problems and the role limitation due to emotional problems domains. Conversely, community-dwelling participants had the best scores in the three domains that the assisted-living participants had their poorest score. The older age, more female gender, more comorbid conditions and less occupational activity recorded among the assisted-living group than those in the community could have resulted to these differences [41].

### FOF among the participants

FOF was very highly prevalent among the older adults in ALFs, and this prevalence was significantly higher than that among community-dwelling participants. This may be associated with their older age, predominance of female gender, lower PA level and presence of more health conditions when compared with their community-dwelling peers. All these had been reported to increase FOF [13, 15, 42, 43]. The near non-existence of FOF among the community-dwelling older adults with just one person that is fearful may not be unconnected with their higher level of PA (as well as their younger age, lesser proportion of females and fewer comorbidities) when compared with their assisted-living counterparts. This may be buttressed by the revealed significant relationships between FOF and PA in both groups of participants in the present study. Association between FOF and poor PA or physical functioning have been previously reported by other authors [13, 44, 45].

### Correlational analysis

Significantly positive correlations exist between FOF and QOL in both groups of participants suggesting that interventions targeted at FOF may have similar effect on the QOL of both groups of participants. The magnitude of the correlation between FOF and QOL in both groups was also similar. Previous studies have reported FOF to be significantly related to QOL among older adults [17, 46, 47]. Physical activity and QOL were significantly and moderately correlated in both groups. Findings of positive relationship between QOL and PA have been previously reported [48–50]. Physical activity more strongly correlated with QOL in the community-dwelling group than the assisted-living group. Though PA will impact positively on the QOL of both groups, it seems that older adults in the community will likely benefit better from PA interventions. This reveals that the interaction between QOL and PA is stronger among community-dwelling older adults than assisted-living older adults. We reasoned that active participation in communal and social life and also the various occupations that serves as their means of livelihood (likely to contribute to PA level) might have further boosted the QOL of the community-dwelling older adults.

The present study also revealed significant relationships between FOF and PA in both groups of participants. The correlation between the two constructs was however stronger in the assisted-living than in the community-dwelling group. Association between FOF and poor PA or physical functioning have been previously reported by other authors [51, 52]. It then seems that PA interventions may benefit older adults in the ALFs more than their peers in the community in terms of moderating FOF. FOF had been reported to curtail PA that may consequently impair the QOL (QOL) of the older adult [17].

### Limitations

Certain limitations of the present study ought to be acknowledged. Present degree of frailty/comorbidities among the participants was not factored into the design of the study and the casual observation of its preponderance in the ALF group suggests that the findings of this study should be interpreted with some caution. The interpretation of the findings is also limited by the cross-sectional nature of the study. Longitudinal design that would ensure assessment of the main outcomes of interest at multiple time points would have given a deeper understanding of the processes and the interactions between PA, FOF and QOL. Participant recruitment into both groups was also not done based on age and gender matching; however, we statistically controlled for these variables to rule out their effects on the observed findings.

### Implications for practice and research

This study provides insight into how participants in the ALF faired and compared to their community-dwelling counterparts in terms of the FOF, PA and QOL. It is also probably one of very rare studies profiling the characteristics of older adults from African populations in ALFs especially as it relates to these health-related constructs. Present results may be suggesting that ageing in place ensures better health outcomes than institutionalised ageing. Hence, whenever possible, older adults should be encouraged to age in place rather than moving into ALFs. However, there is still need for stakeholders in ALFs to improve the living conditions of these facilities considering the fact that ageing in place may be impossible for some older adults. This can be in the form of routine exercises (strengthening, flexibility, balance and coordination), improved social network and psychoeducation aimed at changing negative perception of ALFs. This could be achieved through working together of clinicians, ALFs managers and caregivers. ALF managers need to create an enabling and safe environment for performance of some household and vocational activities among the residents. Clinical services particularly physiotherapy is required at all the ALFs to address some of the comorbidities besetting these older adults. This may help them to be physically active, develop more confidence and have better QOL. Future studies should consider longitudinal design encompassing large range of covariates (like comorbidities, disability level, psychosocial constructs, etc.) in order to better compare the wellness of ageing in place and institutionalised ageing.

## Conclusions

This study was carried out in Nigeria (which is developing country) and thereby provides a unique contribution to the existing body of research. Older adults in the ALFs had lower PA and QOL scores with higher prevalence of FOF than their community-dwelling counterparts, indicating that the latter group move more and feel better than the former group. Significant relationships existed between PA, FOF and QOL for participants in either group, suggesting that physical activity interventions will impact positively on FOF and QOL especially among community-dwelling older adults. Present results may be suggesting that ageing in place ensures better health outcomes than institutionalised ageing.

## Data Availability

The datasets used and/or analysed during the current study are available from the corresponding author on reasonable request.

## Abbreviations

FOF: Fear of falling
QOL: Quality of life
PASE: Physical activity scale for the elderly
MFES: Modified fall efficacy scale
SF-36: Health survey 36-item questionnaire
FES: Fall efficacy scale
SD: Standard deviation
ALF: Assisted-living facility
CD: Community-dwelling
RLPF: Role limitation due to physical problem domain score
RLEP: Role limitation due to emotional problem domain score
MCS: Mental component score
PCS: Physical component score
